# Reply to: Comment on: “Using Field Based Data to Model Sprint Track Cycling Performance”

**DOI:** 10.1186/s40798-021-00351-5

**Published:** 2021-08-23

**Authors:** Hamish Ferguson, Chris Harnish, Geoff Chase

**Affiliations:** 1 Centre for Bioengineering, Department of Mechanical Engineering, University of Canterbury, Private Bag 4800, Christchurch, 8140 New Zealand; 2 Department of Exercise Science, College of Health, Mary Baldwin University, Staunton, VA USA; 3 Centre for Bioengineering, Department of Mechanical Engineering, University of Canterbury, Private Bag 4800, Christchurch, 8140 New Zealand

Dear Editor,

We thank Dr. Douglas [1] for his interest in our literature review outlining our current understanding of sprint cycling [2]. In our review, we use existing data to propose a new, more comprehensive model for sprint cycling performance, to which we recently added more evidence to support this hypothesized model [3]. In his letter [1], Dr. Douglas lays out specific criticisms of our “data” and recommendations by creating a strawman argument. However, much of what he argues against was never actually written in our literature review.

For example, our work [2] never states aerobic training should be prioritized, arguing instead that aerobic training is likely undervalued and a mixture of training may be required. More specifically, our literature review presents a broad swathe of the available data, although not every paper available, as any good review does, and then presents an interpretation and recommendations based on that literature. Specifically, our review makes the case that peak power output (PPO) measures and sprint training alone may limit both assessment and training of sprint cyclists. We also contend sprint cycling should consider the potential contributions of aerobic training to improve performance, and in fact PPO. Dr. Douglas’ fundamental claim is essentially based on the idea any aerobic training limits PPO and thus performance [1]. We feel the data disagree and, equally, state several times more research is needed [2].

This response clarifies aspects of our review which have not been clearly interpreted.

## Peak Power is a Clear Part of Sprint Performance

Our literature review [2] never suggests PPO is not a component of sprint cycling performance, and nor do we assert it should not be an objective of training. Our review highlights the current lack of data beyond piecemeal studies examining individual components of sprinting. Dorel et al. [4] focused on the flying 200-m component of the sprint event, which is one race of 9–12 races over a 1–2-day period. Phillips and Hopkins [5] focused on the relationships between the flying 200-m time and the overall outcomes, which ignores the tactical components of the sprint event. We fail to see how 74% of PPO explains 30 s Wingate test performance, when only correlation analysis was used [6], and compared to data showing counter movement jump, seated Wingate PPO and seated Wingate average power for 30 s only explained 41–66% of performance of the starting straight in BMX [7]. Our use of Figs. 2 and 3 in our review [2] describe the performance envelope of the flying 200-m and of a sprint race, not as anecdote or proof, and it is misleading to state this is our intention as all peer reviewers did not read it this way. More directly, it is clear PPO explains only a large fraction, but far from all, of sprint performance, a gap being addressed in our review.

**Fig. 1:**
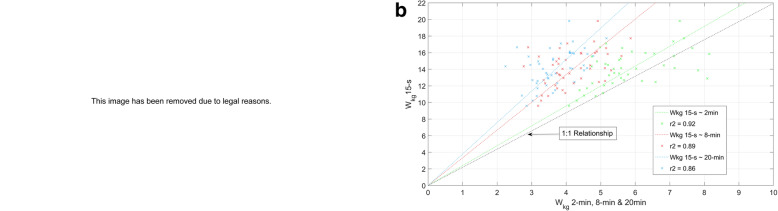
**b** A comparison of 15 s *W*_kg_ with three durations that rely on oxidative energetic supply. All data are from national level sprint cyclists and show very high correlations of *R*^2^ = 0.86–0.92, showing strong inter-relationships of endurance and power

In particular, our review challenges current sprint cycling models solely based on PPO [4, 8]. We recently published data [3] showing a strong relationship between 15-s and 30-s power and power at 2 min, 8 min, and 20 min. In a public debate, instigated by Cycling New Zealand with author HF, Dr. Douglas presented data claiming a near perfect relationship between PPO and 30-s power using a chart based on high performance data [9]. We compared these data to our data and found the claimed relationships were stronger between our data using national level sprinters and the high performance sprinters than the near 1:1 relationship claimed (Fig. 1a). Figure 1b further demonstrates the strong inter-relationship of peak power and oxidative energy system intervals of 2–20 min with *R*^2^ = 0.86–0.92 [3], which are far higher than the correlations of PPO and short period Wingate tests [6, 7]. These very recent results further validate our point that sprint performance is not strictly a function of PPO, whereas if this conjecture of Dr. Douglas’ was true, correlation of PPO would be very low for these longer intervals. It is not.

We refer Dr. Douglas to the many recent works showing the importance of PPO to sprint cycling performance to assure him we are not dismissing the concept [10–13], but are instead moderating it towards a hybrid approach as suggested by the data and relevant literature and analysis in our review.

## Track Cycling Sprinting is Multiple Sprint Performance

We wish to make it clear; we did not refer to track cycling sprinting as repeated sprint exercise [2]. All track cycling sprint events require multiple races in a day. We are not deterred in this area of investigation by a single subject case study that does not present data from all sprints [14], nor by a study which provides no PPO data, and actually supports our contention that the oxidative system becomes increasingly involved in later efforts in the multiple sprint protocol [15]. Our review [2] highlighted the absence of actual data from sprint cycling competition and directed the reader towards studies using a sprint and recovery duration similar to sprint competition, where there were performance drops after a 30–60-min recovery period [16–18].

## Summary

We believe our review [2] summarizes the current state of research and presents a reasonable interpretation, as all good reviews should. Our review argues PPO alone is an insufficient model to account for a track cycling sprint race, let alone a race series involving several races over a day, all of which is supported by data. Our review thus highlights both the well-established critical power model [19] and the anaerobic speed reserve model [20]; suggests, in turn, that these models are not complete; and proposes there may be models better reflecting the physiology and demands of sprint cycling. From this point, we propose a more comprehensive approach may be required to better prepare sprint cyclists, based on the available data, including both PPO and oxidative energy systems training.
