# Correction: Reply to: Comment on: “Using Field Based Data to Model Sprint Track Cycling Performance”

**DOI:** 10.1186/s40798-022-00544-6

**Published:** 2022-12-21

**Authors:** Hamish Ferguson, Chris Harnish, Geoff Chase

**Affiliations:** 1 Centre for Bioengineering, Department of Mechanical Engineering, University of Canterbury, Private Bag 4800, Christchurch, 8140 New Zealand; 2 Department of Exercise Science, College of Health, Mary Baldwin University, Staunton, VA USA

**Correction: Sports Medicine - Open (2021) 7:61** 10.1186/s40798-021-00351-5

After publication of ‘Reply to: Comment on: “Using Field Based Data to Model Sprint Track Cycling Performance”’ [1], the publisher was notified by the data owner that the authors did not have permission to reproduce the data in Fig. 1a, “A comparison of Pmax and watts for 30 s.”.

As a result, Fig. 1a has been removed from the original article.

## References

[1] Ferguson H, Harnish C, Chase G. Reply to: Comment on: “Using Field Based Data to Model Sprint Track Cycling Performance”. Sports Med - Open. 2021;7:61. 10.1186/s40798-021-00351-5. 10.1186/s40798-021-00351-5 PMC8380603 34423390

